# Epidemiology of Tropical Neglected Diseases in Ecuador in the Last 20 Years

**DOI:** 10.1371/journal.pone.0138311

**Published:** 2015-09-22

**Authors:** Monica Cartelle Gestal, Alina Maria Holban, Santiago Escalante, Marcelo Cevallos

**Affiliations:** 1 Department of Microbiology, Secretary for Higher Education, Science, Technology and Innovation of Ecuador, Quito, Ecuador; 2 Department of Microbiology Immunology, Faculty of Biology, University of Bucharest, Bucharest, Romania; 3 Department of Intelligence, Ministry of Public Health, Quito, Ecuador; 4 Department of Economy of Health, Ministry of Public Health, Quito, Ecuador; Albert Einstein College of Medicine, UNITED STATES

## Abstract

**Background:**

Tropical and zoonotic diseases are major problems in developing countries like Ecuador. Poorly designed houses, the high proportion of isolated indigenous population and under developed infrastructure represent a fertile environment for vectors to proliferate. Control campaigns in Ecuador over the years have had varying success, depending on the disease and vectors targeted.

**Aims:**

In our study we analyse the current situation of some neglected diseases in Ecuador and the efficiency of the control campaigns (by measuring changes in numbers of cases reported) that the Ecuadorian government has been running to limit the spread of these infectious and parasitic diseases.

**Results:**

Our study reveals that Brucellosis, Chagas Disease, Rabies and Onchocerciasis have been controlled, but small outbreaks are still detected in endemic areas. Leptospirosis and Echinococcosis have been increasing steadily in recent years in Ecuador since the first records. The same increase has been reported world-wide also. Better diagnosis has resulted in a higher number of cases being identified, particularly with regard to the linking of outdoor activities and contact with farm animals as contributing vectors. Improvements in diagnosis are due to regular professional training, implementation of automatized systems, establishing diagnosis protocols and the creation of an epidemiological vigilance network that acts as soon as a case is reported.

**Conclusion:**

Control campaigns performed in Ecuador have been successful in recent years, although natural phenomena limit their efficiency. Leptospirosis and Echinococcosis infections remain a growing problem in Ecuador as it is worldwide.

## Introduction

There are several clinically and economically important tropical infectious diseases in Ecuador, six of which have been subject to long-standing control campaigns. These six diseases are reportable in the country and include: Chagas Disease (CD), onchocerciasis, rabies, leptospirosis, cystic echinoccocosis (hydatid disease) and brucellosis. The incidence and prevalence of most of these diseases have declined over the last 20 years, owing mainly to the control and surveillance activities implemented by the government through the Ministry of Health. Yet, factors such as climate change, natural disasters, El Nino and other social behaviours have made difficult to completely eliminate these diseases. These diseases have many economic and patient related consequences. There are many causes, for instance, in urban and suburban areas where sewage systems are inadequate, rain water can overwhelm them causing sewage waste to mix with flood water on the surface, increasing the rate of infections by facilitating the proliferation of pathogens and vectors. Among other major factors are inadequate hygiene practices, difficult environmental conditions, farming practices and human activity.

There are several reports regarding the rate and variety of tropical and zoonotic diseases in the most affected South America countries, but few using data from Ecuador and none have been compiled and analysed in such a way as to give a complete overview.

In this paper we discussed the seven most common diseases reported in Ecuador. We have excluded Dengue and Malaria which, due to their importance, are the subject of a separate study (manuscript in preparation).

Chagas disease (CD) caused by *Trypanosoma cruzi* is endemic in Latin America [1]. Infection is normally through Triatomine bugs [2] and worldwide, the incidence is high in rural areas where environmental and socio-economic conditions encourage breeding [3]. In Latin America the more common path is by vectorial transmission (Grijalva et al PLOS NTD 2014) however it is important also to note the path via infected blood transfusion.

Ecuador has been especially vulnerable on both counts due to the way houses are constructed and to the socio-economic situation [3].

While 7 to 8 million people are estimated to be infected with *T*. *cruzi* worldwide, the main concentrations are in Latin America with an estimated 25 million people living in risk areas, in 2008 alone, 10,000 CD- related deaths were reported.

Onchocerciasis (River blindness) is caused by the nematode *Onchocerca volvulus*. The vector of this disease is the Black fly (*Simulium exiguum*). The World Health Organization (WHO) estimates that at least 25 million people are infected with *O*. *volvulus* worldwide and around 123 million people are at risk of becoming infected with the parasite [4]. Ecuador has been specially affected by the disease in the river regions [5–9] but nowadays the disease is controlled.

Rabies is a viral disease present in most regions of the world but approximately 99% of rabies cases are found in developing countries, 90% of these occurring in Asia. Transmission occurs by contact, often a bite, from an infected animal. The first paper reporting bovine rabies in Ecuador was in 1958 [10] and canine rabies in the province of Guayas in 1965 but the first report in the literature about Human rabies in Ecuador was not published until 2010 [11]. Since then, only three papers have been published, two reporting transmission by bats in 2010 [11] and 2012 [12] and one that correlates the serotype of the virus in Ecuador with the rabies virus isolated in Trinidad in 2013 [13].

Leptospirosis is an increasing worldwide problem; the spirochete is transmitted by contact with water contaminated with urine from infected animals. Precise numbers of cases vary between countries but the WHO estimate rates of around 0,1–1 cases per 100 000 inhabitants in temperate regions and is often over 10 times more in tropical regions. Not a lot of literature is found about Leptospirosis in Ecuador apart from a few clinical cases [14], a review of data from the Ministry of Health and OIE presenting cases from Ecuador [15] and a 2011 report of *Leptospira spp*. in the rivers of the rainforest [16], no other reports from Ecuador are found in PubMed. As is the case in many developing countries, much research data remains in the grey literature and even when published, many research papers are published in local journals, not indexed in PubMed, but after a search in local journals and Spanish publications we still did not find a lot of literature about it.

Echinococcosis or Cystic Echinococcosis (CE) are characterized by the formation of cysts on the hosts’ liver or lungs and are caused by tape worm parasites, transmission to humans is normally through ingestion of contaminated meat and water [4]. Around one million people suffer from this disease worldwide [17]. This parasite can be found globally but is endemic in some regions including South America and China [17–21]. Several papers have been published describing this disease in Ecuador [22–24].

Brucellosis (mainly *Brucella abourtus*) is transmitted by contact with infected faeces, urine and milk from infected animals, but most commonly the infection occurs through poorly prepared and/or preserved food of animal origin. Poor personal hygiene practices also contribute to increase incidence. In endemic areas, the reported incidence from data compiled by WHO quotes ranges from less than 0.01 to more than 200 cases per 100,000 inhabitants [4]. In 2013 a paper was published demonstrating significant space-time clustering found in the northern and southern highlands and parts of Ecuadorian Amazonia. In Ecuador in 2014 a study was published which showed that dairy products were a way of transmitting this zoonotic diseases [25, 26]. Also in 2014 a study was published estimating that the seroprevalence is 1.88% in Ecuador in general but higher in rural areas [27].

This paper reviews the general situation regarding these diseases with particular reference to the efficiency of the control campaigns that the Ecuadorian government has been running to limit their spread.

## Materials and Methods

This retrospective study was based on clinical data, gathered from cases reported by medical doctors directly contacting the Epidemiological Surveillance Department of the Ministry of Public Health of Ecuador (14,483,499 million people in 2010, last census). All the data used for this study were approved by the ethical committee of the Ministry of Public Health. A Case was considered, when patients that have been diagnosed and the bacteria, virus or parasite have been isolated and identified. The comparisons made between different areas are based on officially reported cases for each health care district. These data are available on line in the Ministry website (http://www.salud.gob.ec/direccion-nacional-de-vigilancia-epidemiologica/).

The data of the control campaigns were obtained from paper files supplied to the authors by the Economy Department of the Ministry of Public Health of Ecuador[28] (these data are not available online, but can be obtained by contacting the authors).

The control campaigns are focus in the following aspects:

- Study of the epidemiology and distribution of the diseases; to identify where to invest more time and effort and localise hot spots.
- Vigilance of the diseases; taking random samples to look for the bacteria, virus or parasite.
- Cleaning campaigns; to decrease the number of spaces that can accumulate water during the floods and get rid of other dangerous biological material.
- Public education programs; to ensure that the members of public are aware of the causes and effects of the diseases and preventative measures that can be taken at a personal level.
- Training of health care professionals; continuous training for better diagnosis, prognosis and care of the patients.
- Quick response Teams; The setting up of teams to take rapid remedial action after the notification of a case (human or animal).
- Prevention treatments; isolation of human or animal carriers, vaccination if applicable, treatments against vectors and other measures to control the spread.

For the analysis of the data we follow these steps:

(i) Gathering temporal epidemiological data (from website indicated but clarifying the source for data on Chagas and Onchocerciasis); (ii) analysis of the Ministry of Public Health control and surveillance programs; (iii) literature searches from Medline, PubMed and other scientific sources of JCR manuscripts.

## Results and Discussion

While Ecuador still suffers from some vector borne and zoonotic diseases (Table 1), our study shows that Government initiatives have been largely successful in controlling several. The continuous increase of investment in health has improved health services in all areas, resulting in an improvement in diagnosis and better trained professionals, which in turn has shown a decline in most of these diseases. Cofounders such as weather effects, natural phenomena such as earth quakes or volcanos seem to have a minor effect. Two, however, Leptospirosis and Echinococcosis, are still problematic in Ecuador and are an increasing health issue around the world. The limitations of the data are based on the fact that while we have cases reported, indications are that there is a high level of unreported cases meaning that the data are probably underestimates. We do not have the data from the Ministry of Agriculture and Livestock because our study is focused in human cases.

**Table 1:** Number of new cases per year in a population of 14,483,499 million inhabitants over a 20 years period.

	Chagas (In children less than 5 years old)	Onchocerciasis	Rabies	Leptospira	Echinococcosis	Brucellosis
**1994**	42	61	11	0	0	24
**1995**	31	7	20	2	1	13
**1996**	13	10	75	1	2	9
**1997**	9	12	9	3	0	5
**1998**	12	7	7	398	0	1
**1999**	17	4	5	29	0	5
**2000**	8	9	3	63	0	3
**2001**	36	17	3	28	0	5
**2002**	12	53	0	183	0	2
**2003**	25	10	0	155	0	1
**2004**	22	2	0	141	0	4
**2005**	11	0	2	59	0	6
**2006**	20	9	0	122	0	5
**2007**	37	9	0	155	0	9
**2008**	0	0	0	271	21	0
**2009**	9	0	1	212	6	0
**2010**	0	0	0	129	8	0
**2011**	0	0	11	376	14	0
**2012**	0	0	0	1279	14	2
**2013**	0	0	0	894	30	4

This data has been obtained by the Ministry of Public Health of Ecuador from the number of cases reported from each hospital or health centre. This shows the tendency of the NTDs in the last 20 years (Census number has been obtained from the website of INEC http://www.ecuadorencifras.gob.ec/resultados/ where the last data are from 2010).

The control campaigns run by the government of Ecuador have been successful in most cases, taking the numbers reported as an indicator. For all diseases discussed in this manuscript there are standardised protocols for containment and treatment, and are as follows: 1. Passive monitoring that triggers active searches for potential cases, 2. Educational campaigns for dissemination of risk factors and prevention, 3. Isolation of cases, 4. Disinfection of area, 5. Search for the source of infection of index case, 6. Treatment and prophylaxis. However, there are still some zoonotic diseases that continue to be prevalent. Brucellosis is found in low rates in farm animals, usually associated with rural areas. Leptospirosis is increasing in Ecuador (as it is all around the world). It has been associated to a massive increase in intensive livestock farming with its associated levels of effluent, combined with the poor management of the effluent and increased water related outdoor activities such as canoeing and swimming [29].

**Fig 1:**
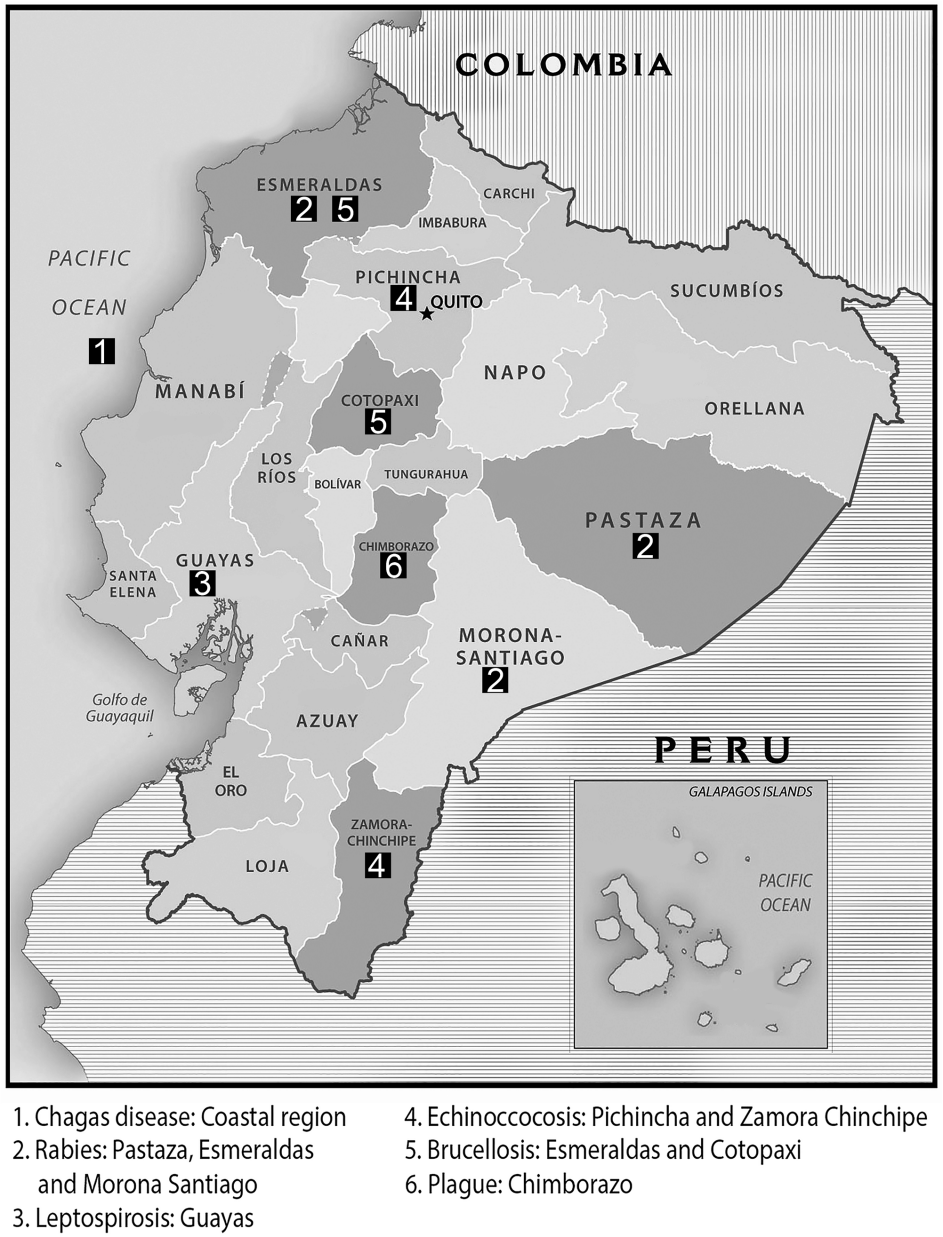
Map of Ecuador indicating outbreaks in provinces. Provinces where outbreaks of tropical and neglected diseases have been reported. 1. Chagas disease: Coastal region, 2. Rabies: Pastaza, Esmeraldas and Morona Santiago, 3. Leptospirosis: Guayas, 4. Echinococcosis: Pichincha and Zamora Chinchipe, 5. Brucellosis: Esmeraldas and Cotopaxi, 6. Plague: Chimborazo. Figure modified from map obtained from www.freemapviewer.com (copyright public domain).

In Ecuador, Chagas Disease and Onchocerciasis have all but disappeared. Since 2007 no cases have been reported, except a small outbreak in 2009 where a total of 9 people were diagnosed with Chagas disease, 7 of them in the coast region (Fig 1). The control campaigns have reduced the level of these two diseases to zero since 2010 for Chagas Disease and 2008 for Onchocerciasis and the effect has been maintained so far, indicating that these two diseases appear to have been effectively controlled (Table 1).

Chagas Disease: In wider Latin America there have been important successes in parasite and vector control since the 1990s in the intergovernmental territories of the Southern Cone, Central American, Andean Pact and Amazonian regions. These multinational initiatives have led to substantial reductions in transmission by vectors. In addition, the risk of transmission by blood transfusion has been substantially reduced by the strict testing of blood donors with ELISA before the extraction, and better control of the storage and use of blood [1–3]. Other control measures include encouragement to improved design of houses, use of mosquito nets and discouragement of bringing animals in to the house, especially in the more effected regions of the coast and amazon.

The programs for the control of CD in Ecuador started in 2003, now, a decade later, the disease is considered controlled. Our results show that from 2010 no new cases were reported in children less than five years old, indicating that the program of vector vigilance and control of blood transfusion practices are effective in the control of this disease[3].

The Onchocerciasis (*Onchocera volvulus*) Elimination Program of the Americas was established in 1992 in response to a resolution of the Directing Council of the Pan American Health Organization (PAHO) calling for the elimination of Onchocerciasis Ocular Morbidity in the Americas by 2007 [30]. This disease has been considered eliminated by the WHO [4] following initiatives by the Ecuadorian government that when a case is reported the local inhabitants are encouraged to cover up and avoid being bitten. When an outbreak is suspected the government sprays the risk area with Ivermectin.

In 2007 Lindblade [31] conducted an evaluation of *O*. *volvulus* transmission in Santa Rosa, Guatemala, guided by criteria developed by the WHO. The authors found no evidence of current infection or recent transmission and they encountered no seropositive children between the ages of 6 and 12 years. Britan et al, in 2009 reported rates of Onchocerciasis of 6.5% in Brazil, <0.1% Ecuador, 0.4% Guatemala, 0.1% Mexico and 5% Venezuela. The same results were published later on, confirming the status of onchoccerciasis as controlled [32]. In a paper published in 2011, the author showed data that in Venezuela and Brazil there are areas where onchocerciasis is still prevalent, in Colombia, Mexico and Ecuador was considered interrupted and in Guatemala was considered supressed [7]. However, onchocerciasis in Central America is still a threat, where less than 1% is affected but 41% of the population was at risk of being infected. Guatemala had the largest number of people living in onchocerciasis endemic regions (231,000) [33].

In 2013 Colombia was awarded by the WHO for the elimination of Onchocerciasis followed by Ecuador in 2014, Mexico and Guatemala are still in post-treatment vigilance and if the results are good, both countries can be awarded with the certificate of elimination of Onchocerciasis in 2015. Still there is an areas in the Amazon region that is still not controlled, Yanomami between Brazil and Venezuela, this areas is a threat to the vision of the whole continent free of this disease [34].

Regarding Ecuador, in 2007, Vieira et al [9], showed that in 2004 there were 5 people in Rio Capayas with skin snips typical of this disease (although the Epidemiological Surveillance Department reported just 2 cases in that year) but the report concluded that while the disease had been controlled in most of the country by 2007, the authors considered that the failure in the Rio Capayas area indicated that a change of strategy was needed. The strategy they suggested was an intensive program of increased frequency of Ivermectin (Mectizan MT) treatment in combination with a course of Doxycycline, this would sterilize the adult female flies (the main vector), and yield rapid results in the suppression of transmission of *O*. *volvulus*.

In another paper published by Guevara et al in 2002 [6] they studied a wide batch of flies collected from endemic areas of Ecuador and observed that the transmission rate decreased drastically after six semi-annual treatments with Ivermectin.

As a consequence of these programs, Ecuador became the second country to become free of Onchocerciasis with no cases being reported since 2007 [7].

Human Rabies has been largely under control in Ecuadorian cities from 2001, thanks to the Government’s control campaigns, however Rabies proved more difficult to control in rural areas because of wild animals and the problem with transmission via bat bites is a difficult if not impossible problem to solve due to outbreaks being located in the rain forest. During the control campaigns the government vaccinated all the dogs (domestic and wild) in the cities; it ran educational campaigns, vaccination campaign for humans in contact with suspicious dogs and other control measures like attempting to isolate affected areas. Massive animal vaccination campaigns that the Government ran effectively controlled rabies transmitted by dogs, but it is more difficult when transmission is caused by other wild animals, particularly bats in the Amazon region. In 2005, in Pastaza (Amazon region), there were 2 cases reported, caused by bites from bats. In 2009, In Esmeraldas (Coastal region), 1 case was reported as being transmitted by a cat that carried the virus. Finally, in 2011, in Morona Santiago (Amazon region), 11 cases were reported, again with bats transmitting the disease through their bites (Table 1, Fig 1).

Rabies Ecuador is part of the “Regional plan of Rabies of Americas” driven by the WHO. In 1996, Ecuador registered one of the biggest outbreaks, with 1700 dogs and 75 humans infected, of which 65 died. Since then, the number of cases reported decreased until 2001, when just one case of human rabies transmitted by a dog was reported and apart from the isolated out breaks of 2005, 2009 and 2011, there have been no reports of human rabies (Fig 1). However, human rabies transmitted by dogs is still reported in some Latin American countries, particularly Bolivia, Brazil, Guatemala, Haiti and Peru [35].

In several parts of the world the control of rabies has been successful. In Romania after the outbreak in 2008 the number of cases of rabies reported has been decreasing, with just 6 cases reported between 2008–2012 [36]. In Poland, in 1993 the implementation of the oral vaccination of wild animals was introduced. Since then, a 160 cases where reported in 2011, most being reported in dogs and just one in a fox [37].

Leptospirosis (*Leptospira spp*.) has been increasing in the last few years. In an outbreak observed in 1998, 398 cases (Table 1) were reported; “El Niño” (Southern Oscillation) occurred in the same year with its associated increase in static water and temperature. After the outbreak of 1998 the number of cases has remained relatively low, except in 2011 and 2012 when respectively 376 and 1279 cases were reported, coinciding with “La Niña” (Northern Oscillation). In 2012 it was reported that the source of the outbreak in Rios and Guayas (Coastal Region) was static pond water containing faecal waste and the 2011 outbreak was reported to be due to water contaminated with rat faecal matter. In 2013 there were 894 cases reported, showing a marginal decrease in infection rates. Most reported patients lived close to rivers or near to the coast area. Manabí is the region generally worst afflicted. In 2005 the number of cases decreased drastically, but then in 2006 the number of cases increased from 56 to 122. Between 2006 and 2010 the numbers gradually increased to 376 but in 2011 the number of reported cases more than trebled to 1279 (Fig 1).

Prevalence of Leptospirosis is increasing not just in Latin America but also in the rest of the world. It is especially a problem in developing countries and tropical regions, where more than 500.000 cases are reported every year [38]. The predominance of clinical cases of leptospirosis in men is well recognised [29, 38] and has been explained by their greater tendency to participate in outdoor activities, putting them at high risk of exposure [38]. Similarly, case rates among adults between 20 and 50 years of age are also consistently the highest reported [38, 39].

Ecuador, in line with the rest of the world, is recording an increase of cases of Leptospirosis, possibly because of better diagnostic procedures but also because of the increasing use of intensive livestock farming techniques, poor management of effluent and poor hygiene, all of which lead to increases in infection rates. For those reasons it is mandatory to have personnel trained for the diagnosis, care and treatment of the leptospirosis that can act immediately when needed. Control measures when a case is presented, is to investigate the source of contamination and when identified a clean-up operation is conducted and all those at risk in the area are vaccinated. Information is also disseminated to the population.

Cystic echinococcosis outbreaks were reported in 1995 and 1996, both in the Pichincha region of Ecuador (Fig 1). Between 1997 and 2007 no cases were reported, then in 2008, 21 cases were reported and since then the number of isolates of *Echinococcus spp*. have been increasing (Table 1). Cystic echinococcosis is a growing problem in Ecuador; reports have been increasing, possibly due to better diagnosis, more sensitive methods and better understanding of the disease which makes doctors more alert. However, increases in intensive farming and its associated effluent may contribute to increases. By 2008 infections started to be reported in different areas of Ecuador and reports have continued to increase since then [4]. A human infected with *E*. *vogeli* was reported in Esmeraldas (coastal region) in 1993 [40] but the presence of this specie had been described previously in Ecuador in 1979 [41]. When trying to determine the specie, little information has been register with the Ministry of Public Health. One study published in 2010, Cuenca, showed a prevalence of *E*. *granulosus* in 13.53% of pigs slaughtered in the abattoir [42], the same specie was reported in 2011 in Chimborazo (both Andean regions), finding a prevalence of 2.37% in sheep slaughtered [43]. The same year, this specie was also reported in Puyo (at an altitude of just under 1000 meters) which lies within the Amazonian region closes to the boarder of the Andean region [44]. In 2010, Ibarra (Andean region), a prevalence of 3.87% of *Echinococcus spp*. was found, but no data on specie was registered [45]. It is important to highlight the fact that data are limited and of poor quality, the references that were obtained were mostly Thesis degree works conducted in slaughter houses, which means they are punctual studies in a small geographic regions; on a national level the prevalence is unknown.

In 2012 in Europe most echinococcosis cases were reported in Bulgaria [20], but there are also affected regions in Spain and Italy, in particular Southern Italy and Sardinia [19]. Switzerland and Poland have also seen an increase of echinococcosis linked to an increase in the fox population. In Lithuania the increase in numbers of cases reported has been related with dogs. However, the fact that incidences of alveolar echinococcosis are rapidly increasing could be due to improvements in diagnosis or socioeconomic changes affecting living conditions and life style [21].

Brucellosis (*Brucella spp*.) infection rates have remained relatively constant and low in Ecuador (Table 1) during most of the study period. In 2008 this disease was thought to have disappeared completely, but in 2012, 2 cases were reported in Esmeraldas (Coastal region) and Cotopaxi (Andes) and again in 2013 there were 4 cases reported, 1 in Orellana (Amazonian region), 1 in Napo (Amazonian region) and 2 in Pichincha (Andes) (Fig 1). It is estimated that each year more than 500.000 new cases of Brucellosis occur worldwide, but in Ecuador there have been only a very few cases reported since 2008. This is attributable to the extensive use of vaccines and the general success of governmental campaigns to control the disease and keep rates low [46]. In 2008 the government of Ecuador started a campaign to control bovine brucellosis; the success is shown in the number of cases reported since then, which was zero except in 2012 and 2013. Serological controls are performed on a regular basis in groups of high risk like farm and slaughterhouse workers that are in direct contact with animals that potentially could be infected. When an isolate is discovered animals and humans alike are vaccinated, if treatment of the animal is unsuccessful it is then destroyed. If an infected dairy animal is discovered, the produce from that farm is removed from the market.

Before the government implemented control measures, a study was published [47] regarding the rates of *Brucella spp*. infections in Latin America, between 1968 and 2006 showing that Ecuador had the third lowest number of isolates after the Dominican Republic and Nicaragua. However, countries like Mexico, Argentina and Peru prove to have among the highest prevalence. That said, the situation in Latin America is not as bad as in other regions, for example Niger, where it is proving to be a serious problem, especially in rural areas like Niamey [48].

In China, *Brucella sp*. has been found in several wild animals species [49], whereas in Israel, infection is often through contaminated camel milk [50].

## Conclusions

The Ecuadorian government has been expending increasing amounts of budget in the control of tropical and neglected diseases. The Ecuadorian government has mounted successful control campaigns against most of the tropical and zoonotic diseases described. It was one of the first countries to control Onchocerciasis and Chagas disease, while Rabies has been well controlled except for some small outbreaks caused by wild animals, particularly bats, in the Amazonian region. Brucellosis is well controlled in Ecuador through Governmental initiatives and the number of cases reported continues to be low. Plague is not considered a major issue in Ecuador as all the cases reported are confined to the province of Chimborazo, where the total of 23 cases was in three outbreaks which were successfully treated. No cases have been reported since 2001. This is the reason why we think it is not relevant to highlight as one of the major diseases in Ecuador. Leptospirosis and Echinococcosis are still problems that the government are trying to control through existing initiatives.

The strength of these programs relies on multidisciplinary approaches covering education of the population to stop the dissemination of the diseases; training of professionals to improve the diagnosis and control of the diseases; vaccination or personal risk reduction measures; physical cleaning and hygiene and vector or animal control. The figures indicate the efficiency of the Ecuadorian control campaigns and education programs, and the authors suggest that the approaches can be used as a model for other endemic countries that are still suffering these diseases.

However, it is crucial these diseases should continue to be monitored as, although this research shows positive results, they are endemic to Ecuador. The starting of “El Nino” with its associated effects on proliferation of vectors and increases in contaminated flood waters, offers an ideal opportunity to stress test these initiatives to ensure the efficiency of these control programs.

## References

[1] Pérez-Morales D, Lanz-Mendoza H, Hurtado G, Martínez-Espinosa R, Espinoza B. Proteomic analysis of Trypanosoma cruzi epimastigotes subjected to heat shock. J Biomed Biotechnol. 2012;2012:902803 10.1155/2012/902803 22287837 PMC3263753

[2] Pereira PC, Navarro EC. Challenges and perspectives of Chagas disease: a review. J Venom Anim Toxins Incl Trop Dis. 2013;19(1):34 10.1186/1678-9199-19-34 24354455 PMC3898031

[3] Abad-Franch F, Aguilar M. Control de la enfermedad de Chagas en el Ecuador In: Aguilar M, editor. Quito: OPS/OMS Ministerio de Salud Publica; 2003.

[4] Organization WH. WHO. Available: http://www.who.int/en/.

[5] Cupp EW, Duke BO, Mackenzie CD, Guzmán JR, Vieira JC, Mendez-Galvan J, et al The effects of long-term community level treatment with ivermectin (Mectizan) on adult Onchocerca volvulus in Latin America. Am J Trop Med Hyg. 2004;71(5):602–7. 15569792

[6] Guevara AG, Vieira JC, Lilley BG, López A, Vieira N, Rumbea J, et al Entomological evaluation by pool screen polymerase chain reaction of Onchocerca volvulus transmission in Ecuador following mass Mectizan distribution. Am J Trop Med Hyg. 2003;68(2):222–7. 12641415

[7] Gustavsen K, Hopkins A, Sauerbrey M. Onchocerciasis in the Americas: from arrival to (near) elimination. Parasit Vectors. 2011;4:205 10.1186/1756-3305-4-205 22024050 PMC3214172

[8] Moncayo AL, Vaca M, Amorim L, Rodriguez A, Erazo S, Oviedo G, et al Impact of long-term treatment with ivermectin on the prevalence and intensity of soil-transmitted helminth infections. PLoS Negl Trop Dis. 2008;2(9):e293 10.1371/journal.pntd.0000293 18820741 PMC2553482

[9] Vieira JC, Cooper PJ, Lovato R, Mancero T, Rivera J, Proaño R, et al Impact of long-term treatment of onchocerciasis with ivermectin in Ecuador: potential for elimination of infection. BMC Med. 2007;5:9 17521449 10.1186/1741-7015-5-9 PMC1890547

[10] Bruno Moggia J. [Incidence of canine rabies in Guayas Province and some aspects of its clinical and laboratory diagnosis]. Rev Ecuat Hig Med Trop. 1965;22(2):189–200. 5890784

[11] Castilho JG, Carnieli P, Durymanova EA, Fahl WeO, Oliveira ReN, Macedo CI, et al Human rabies transmitted by vampire bats: antigenic and genetic characterization of rabies virus isolates from the Amazon region (Brazil and Ecuador). Virus Res. 2010;153(1):100–5. 10.1016/j.virusres.2010.07.012 20637811

[12] Lee DN, Papeş M, Van den Bussche RA. Present and potential future distribution of common vampire bats in the Americas and the associated risk to cattle. PLoS One. 2012;7(8):e42466 10.1371/journal.pone.0042466 22900023 PMC3416852

[13] Seetahal JF, Velasco-Villa A, Allicock OM, Adesiyun AA, Bissessar J, Amour K, et al Evolutionary history and phylogeography of rabies viruses associated with outbreaks in Trinidad. PLoS Negl Trop Dis. 2013;7(8):e2365 10.1371/journal.pntd.0002365 23991230 PMC3749974

[14] Valarezo-Sevilla D, Sarzosa-Terán V. [Leptospirosis: case-series report in a prison of the coast in Ecuador]. Rev Esp Sanid Penit. 2014;16(1):20–3. 10.4321/S1575-06202014000100004 24615374

[15] Costa F, Martinez-Silveira MS, Hagan JE, Hartskeerl RA, Galvão dos Reis M, A IK. Surveillance for leptospirosis in the Americas, 1996–2005: a review of data from ministries of health. Revista Panamericana de Salud Publica. 2012;32(3). 10.1590/s1020-49892012000900001 PMC3970205 23183556

[16] Barragan VA, Mejia ME, Trávez A, Zapata S, Hartskeerl RA, Haake DA, et al Interactions of leptospira with environmental bacteria from surface water. Curr Microbiol. 2011;62(6):1802–6. 10.1007/s00284-011-9931-3 21479795

[17] Otero-Abad B, Torgerson PR. A systematic review of the epidemiology of echinococcosis in domestic and wild animals. PLoS Negl Trop Dis. 2013;7(6):e2249 10.1371/journal.pntd.0002249 23755310 PMC3674998

[18] Atkinson JA, Williams GM, Yakob L, Clements AC, Barnes TS, McManus DP, et al Synthesising 30 years of mathematical modelling of Echinococcus transmission. PLoS Negl Trop Dis. 2013;7(8):e2386 10.1371/journal.pntd.0002386 24009786 PMC3757076

[19] Petrone L, Cuzzi G, Colace L, Ettorre GM, Busi-Rizzi E, Schininà V, et al Cystic echinococcosis in a single tertiary care center in Rome, Italy. Biomed Res Int. 2013;2013:978146 10.1155/2013/978146 24151631 PMC3789360

[20] Stoianov G, Iarŭmov N, Petrov D, Kalinova K. 112 years of hydatid surgery in Bulgaria (1895–2007). Khirurgiia (Sofiia). 2007(5):53–7. 18580835

[21] Usubalieva J, Minbaeva G, Ziadinov I, Deplazes P, Torgerson PR. Human alveolar echinococcosis in Kyrgyzstan. Emerg Infect Dis. 2013;19(7):1095–7. 10.3201/eid1907.121405 23763935 PMC3713972

[22] Bruno Moggia J. Hydatidosis in Ecuador. Rev Ecuat Hig Med Trop. 1966;23(3):259–66. 6011798

[23] Carabin H, Torgerson PR, Budke C, Cowan LD, Nash T, Willingham AL, et al Assessing the burden of Taenia solium cysticercosis and echinococcosis. Vet Parasitol. 2004;125(1–2):183–202. 24937886 10.1016/j.vetpar.2004.05.013

[24] Lazo RF. Hydatidosis: human and medical exposition. 1st seminar on zoonosis. Rev Ecuat Hig Med Trop. 1977;30(3):305–13. 384482

[25] Ron-Román J, Saegerman C, Minda-Aluisa E, Benítez-Ortíz W, Brandt J, Douce R. First report of orchitis in man caused by Brucella abortus biovar 1 in Ecuador. Am J Trop Med Hyg. 2012;87(3):524–8. 10.4269/ajtmh.2012.11-0341 22826490 PMC3435359

[26] Ron-Román J, Ron-Garrido L, Abatih E, Celi-Erazo M, Vizcaíno-Ordóñez L, Calva-Pacheco J, et al Human brucellosis in northwest Ecuador: typifying Brucella spp., seroprevalence, and associated risk factors. Vector Borne Zoonotic Dis. 2014;14(2):124–33. 10.1089/vbz.2012.1191 24410144

[27] Herrick JA, Lederman RJ, Sullivan B, Powers JH, Palmore TN. Brucella arteritis: clinical manifestations, treatment, and prognosis. Lancet Infect Dis. 2014;14(6):520–6. 10.1016/S1473-3099(13)70270-6 24480149 PMC4498663

[28] Ecuador MoPHo. Manual De Procedimientos Del Subsistema Alerta Acción Sive–Alerta. Quito, Ecuador: Ministerio de Salud Publica; 2013. 235 p.

[29] Radl C, Müller M, Revilla-Fernandez S, Karner-Zuser S, de Martin A, Schauer U, et al Outbreak of leptospirosis among triathlon participants in Langau, Austria, 2010. Wien Klin Wochenschr. 2011;123(23–24):751–5. 10.1007/s00508-011-0100-2 22105111

[30] Monroy J, Lovato R, Barrera S, Acosta P. Onccocercosis in Ecuador, country report Quito, Ecuador: Ministry of Public Health; 2013.

[31] Lindblade KA, Arana B, Zea-Flores G, Rizzo N, Porter CH, Dominguez A, et al Elimination of Onchocercia volvulus transmission in the Santa Rosa focus of aGuatemala. Am J Trop Med Hyg. 2007;77(2):334–41. 17690408

[32] Cruz-Ortiz N, Gonzalez RJ, Lindblade KA, Richards FO, Sauerbrey M, Zea-Flores G, et al Elimination of Onchocerca volvulus Transmission in the Huehuetenango Focus of Guatemala. J Parasitol Res. 2012;2012:638429 10.1155/2012/638429 22970346 PMC3432545

[33] Hotez PJ, Woc-Colburn L, Bottazzi ME. Neglected tropical diseases in Central America and Panama: review of their prevalence, populations at risk and impact on regional development. Int J Parasitol. 2014;44(9):597–603. 10.1016/j.ijpara.2014.04.001 24846528

[34] Kenyon Aulta S, Catalá Pascual L, Grados-Zavala ME, Gonzálvez García G, Castellanos Lg. El Camino a la Eliminación: un Panorama delas Enfermedades Infecciosas Desatendidasen América Latina y el Caribe. Rev Peru Med Exp Salud Publica. 2014;31((2)):319–25. 25123873

[35] Beran GW, Frith M. Domestic animal rabies control: an overview. Rev Infect Dis. 1988;10 Suppl 4:S672–7. 3206079 10.1093/clinids/10.supplement_4.s672

[36] Najar H, Streinu-Cercel A. Epidemiological management of rabies in Romania. Germs. 2012;2(3):95–100. 10.11599/germs.2012.1019 24432269 PMC3882854

[37] Sadkowska-Todys M, Kucharczyk B. Rabies in Poland in 2011. Przegl Epidemiol. 2013;67(3):473–6, 571–3. 24340563

[38] Poeppl W, Orola MJ, Herkner H, Müller M, Tobudic S, Faas A, et al High prevalence of antibodies against Leptospira spp. in male Austrian adults: a cross-sectional survey, April to June 2009. Euro Surveill. 2013;18(25). 10.2807/1560-7917.es2013.18.25.20509 23806296

[39] Pappas G, Papadimitriou P, Siozopoulou V, Christou L, Akritidis N. The globalization of leptospirosis: worldwide incidence trends. Int J Infect Dis. 2008;12(4):351–7. 18055245 10.1016/j.ijid.2007.09.011

[40] datos Pebd. Hidatidosis en el Ecuador: informe del undécimo caso echinococcus vogeli / Hidatidosis in Ecuador: report of the eleventh case echinococcus vogeli http://bases.bireme.br/cgi-bin/wxislind.exe/iah/online/?IsisScript=iah/iah.xis&src=google&base=LILACS〈=p&nextAction=lnk&exprSearch=235267&indexSearch=ID 1993.

[41] D'Alessandro A, Rausch RL, Cuello C, Aristizabal N. Echinococcus vogeli in man, with a review of polycystic hydatid disease in Colombia and neighboring countries. Am J Trop Med Hyg. 1979;28(2):303–17. 572148 10.4269/ajtmh.1979.28.303

[42] Silverio Allaico G, Patricio Jimenez C. Determinacion de Hidatidosis en cerdos faenados en el Camal de Azogues Cuenca: Universidad de Cuenca. Facultad de Ciencias Agropacuarias. Escuela de Medicina Veterniaria y Zootecnia; 2010.

[43] Zambrano G, Paulina S. Estudio sanitario-productivo de la afeccion endoparasitaria por cestodos en ovinos mestizos Escuela Superior Politecnica del Chimborazo: Escuela Superior Politecnica del ChimborazoFacultad de Ciencias PecuariasEscuela de Ingenieria Zootecnica; 2011.

[44] Torres Andrade FA. Identificación de la presencia de Hidatidosis en el Camal Municipal de la ciudad de Puyo, Provincia de Pastaza Quito, Ecuador: Universidad Central Del Ecuadorfacultad De Medicina Veterinaria Y Zootecniacarrera De Medicina Veterinaria Y Zootecnia; 2012.

[45] Chasiquiza T, Elizabeth M. Determinacion de la incidencia de Hidiatosis porcina *Echinococcus sp*. en los animales faenados en la empresa municipal del rastro Ibarra y el efecto economico en la comercializacion de la carne Escuela Superior Politecnica del Chimborazo: Escuela Superior Politecnica del ChimborazoFacultad de ciencias PecuariasEscuela de Ingenieria Zootecnica; 2010.

[46] Manias V, Nagel A, Mollerach A, Mendosa MA, Freyre H, Gómez A, et al Brucella canis endocarditis: first documented case in Argentina. Rev Argent Microbiol. 2013;45(1):50–3. 23560789

[47] Cabanel N, Leclercq A, Chenal-Francisque V, Annajar B, Rajerison M, Bekkhoucha S, et al Plague outbreak in Libya, 2009, unrelated to plague in Algeria. Emerg Infect Dis. 2013;19(2):230–6. 10.3201/eid1902.121031 23347743 PMC3559055

[48] Dean AS, Bonfoh B, Kulo AE, Boukaya GA, Amidou M, Hattendorf J, et al Epidemiology of brucellosis and q Fever in linked human and animal populations in northern togo. PLoS One. 2013;8(8):e71501 10.1371/journal.pone.0071501 23951177 PMC3741174

[49] Luo J, Zeng Z, Song Y, He H. Brucellosis in takins, China. Emerg Infect Dis. 2012;18(9):1527–9. 10.3201/eid1809.120069 22931790 PMC3437706

[50] Shimol SB, Dukhan L, Belmaker I, Bardenstein S, Sibirsky D, Barrett C, et al Human brucellosis outbreak acquired through camel milk ingestion in southern Israel. Isr Med Assoc J. 2012;14(8):475–8. 22977965

